# Characterization of MxiE- and H-NS-Dependent Expression of *ipaH7.8*, *ospC1*, *yccE*, and *yfdF* in Shigella flexneri

**DOI:** 10.1128/msphere.00485-22

**Published:** 2022-11-08

**Authors:** Chelsea P. Hall, Niti B. Jadeja, Natalie Sebeck, Hervé Agaisse

**Affiliations:** a Department of Microbiology, Immunology, and Cancer Biology, University of Virginia School of Medicine, Charlottesville, Virginia, USA; University of Kentucky

**Keywords:** H-NS, MxiE, *Shigella*, T3SS, anti-silencing, silencing

## Abstract

Shigella flexneri uses a type 3 secretion system (T3SS) apparatus to inject virulence effector proteins into the host cell cytosol. Upon host cell contact, MxiE, an S. flexneri AraC-like transcriptional regulator, is required for the expression of a subset of T3SS effector genes encoded on the large virulence plasmid. Here, we defined the MxiE regulon using RNA-seq. We identified virulence plasmid- and chromosome-encoded genes that are activated in response to type 3 secretion in a MxiE-dependent manner. Bioinformatic analysis revealed that similar to previously known MxiE-dependent genes, chromosome-encoded genes *yccE* and *yfdF* contain a regulatory element known as the MxiE box, which is required for their MxiE-dependent expression. The significant AT enrichment of MxiE-dependent genes suggested the involvement of H-NS. Using a dominant negative H-NS system, we demonstrate that H-NS silences the expression of MxiE-dependent genes located on the virulence plasmid (*ipaH7*.8 and *ospC1*) and the chromosome (*yccE* and *yfdF*). Furthermore, we show that MxiE is no longer required for the expression of *ipaH7.8*, *ospC1, yccE*, and *yfdF* when H-NS silencing is relieved. Finally, we show that the H-NS anti-silencer VirB is not required for *ipaH7.8* and *yccE* expression upon MxiE/IpgC overexpression. Based on these genetic studies, we propose a model of MxiE-dependent gene regulation in which MxiE counteracts H-NS-mediated silencing.

**IMPORTANCE** The expression of horizontally acquired genes, including virulence genes, is subject to complex regulation involving xenogeneic silencing proteins, and counter-silencing mechanisms. The pathogenic properties of Shigella flexneri mainly rely on the acquisition of the type 3 secretion system (T3SS) and cognate effector proteins, whose expression is repressed by the xenogeneic silencing protein H-NS. Based on previous studies, releasing H-NS-mediated silencing mainly relies on two mechanisms involving (i) a temperature shift leading to the release of H-NS at the *virF* promoter, and (ii) the virulence factor VirB, which dislodges H-NS upon binding to specific motifs upstream of virulence genes, including those encoding the T3SS. In this study, we provide genetic evidence supporting the notion that, in addition to VirB, the AraC family member MxiE also contributes to releasing H-NS-mediated silencing in S. flexneri.

## INTRODUCTION

*Shigella* spp. are human-specific bacterial pathogens which are the causative agents of bacillary dysentery, also known as bloody diarrhea (1). Transmission occurs via the fecal-oral route and requires a very low infectious dose to cause disease (2). The disease is caused by invasion of the colonic epithelium and bacterial cell-to-cell spread, leading to the destruction of the mucosa, vascular lesions, and massive inflammation (3, 4). *Shigella* spp., as well as other Gram-negative bacteria, utilize a type 3 secretion system (T3SS) needle-like apparatus to directly inject bacterial virulence effector proteins into the host cell cytosol (5). The translocated effector proteins are necessary for virulence through their roles in host-cell invasion, cell-to-cell spread, and modulation of host-cell signaling to promote immune evasion (6).

Descended from commensal Escherichia coli, Shigella flexneri horizontally acquired its AT-rich virulence plasmid, which encodes the proteins necessary for virulence (7–9). The presence of a global repressor known as histone-like nucleoid structuring protein (H-NS, previously referred to as VirR), that preferentially binds AT-rich DNA, is thought to have allowed for the acquisition and maintenance of the energetically costly virulence genes (10, 11). Expression of the S. flexneri virulence genes follows a three-tiered regulatory cascade involving the transcriptional regulators VirF, VirB, and MxiE (12).

At 30°C, AT-rich genes, including those on the virulence plasmid, are silenced by H-NS (13–18). A shift in temperature to 37°C results in a change in DNA topology, which leads to the de-repression of the H-NS-bound *virF* promoter (11, 19, 20). Following *virF* expression at 37°C, VirF, the first-tier regulator, activates the expression of *icsA*, the gene required for actin-based motility and cell-to-cell spread, and *virB*, which encodes the second-tier regulator (17, 21, 22). VirB acts as an intermediate regulator of transcription, overcoming H-NS-mediated transcriptional silencing at a subset of promoters (11, 13–15, 23–28). Genes whose expression relies on VirB include the genes encoding the T3SS, genes encoding virulence factors (*icsP*, *ospZ*, and *ospD1*), and genes coding for the third-tier transcriptional regulator, MxiE, and its coactivator, IpgC (12, 13, 27–30).

MxiE, as well as VirF, are members of the family of AraC-like transcriptional regulators, which are characterized by their DNA-binding domain (DBD) comprised of two helix-turn-helix (HTH) motifs that are thought to bind to the major groove of DNA (31). When the T3SS is not active, MxiE is bound by OspD1, which functions as an anti-activator, and the chaperone of OspD1, Spa15 (32). Meanwhile, IpgC is functioning as the chaperone for the translocon proteins, IpaB and IpaC (33). Upon host cell contact, IpaB and IpaC form the T3SS pore into the host cell membrane and OspD1 is secreted along with the other first wave of effector proteins (28, 34–37). This frees MxiE, which then interacts with its coactivator IpgC, leading to the expression of MxiE-dependent genes (12, 38–40). MxiE-dependent gene expression relies on the presence of a 17-bp *cis*-regulatory element, termed the MxiE box, located in the promoter region of MxiE-dependent genes (41, 42). Although MxiE and IpgC copurify as a complex, evidence that MxiE binds the MxiE box and functions as a transcriptional activator *in vitro* is lacking (39).

In this study, we use RNA-seq to determine the MxiE regulon. In addition to known MxiE-dependent genes located on the large virulence plasmid, we find MxiE-dependent genes located on the chromosome, including *yccE* and *yfdF*, and we demonstrate the functionality of bioinformatically identified MxiE box sequences in their promoter regions (43). Additionally, we demonstrate that H-NS silences the expression of MxiE-dependent genes located on the large virulence plasmid (*ipaH7.8* and *ospC1*) and the chromosome (*yccE* and *yfdF*). Importantly, our genetic studies show that MxiE is no longer required for the expression of *ipaH7.8*, *ospC1*, *yccE*, or *yfdF* when H-NS silencing is relieved. Finally, we show that the anti-silencer VirB is not required for *ipaH7.8* and *yccE* expression upon MxiE/IpgC overexpression. Based on these genetic studies, we propose a model of MxiE-dependent gene regulation in which MxiE counteracts H-NS-mediated silencing.

## RESULTS

### Characterization of the *mxiE*ΔDBD strain.

**FIG 1:**
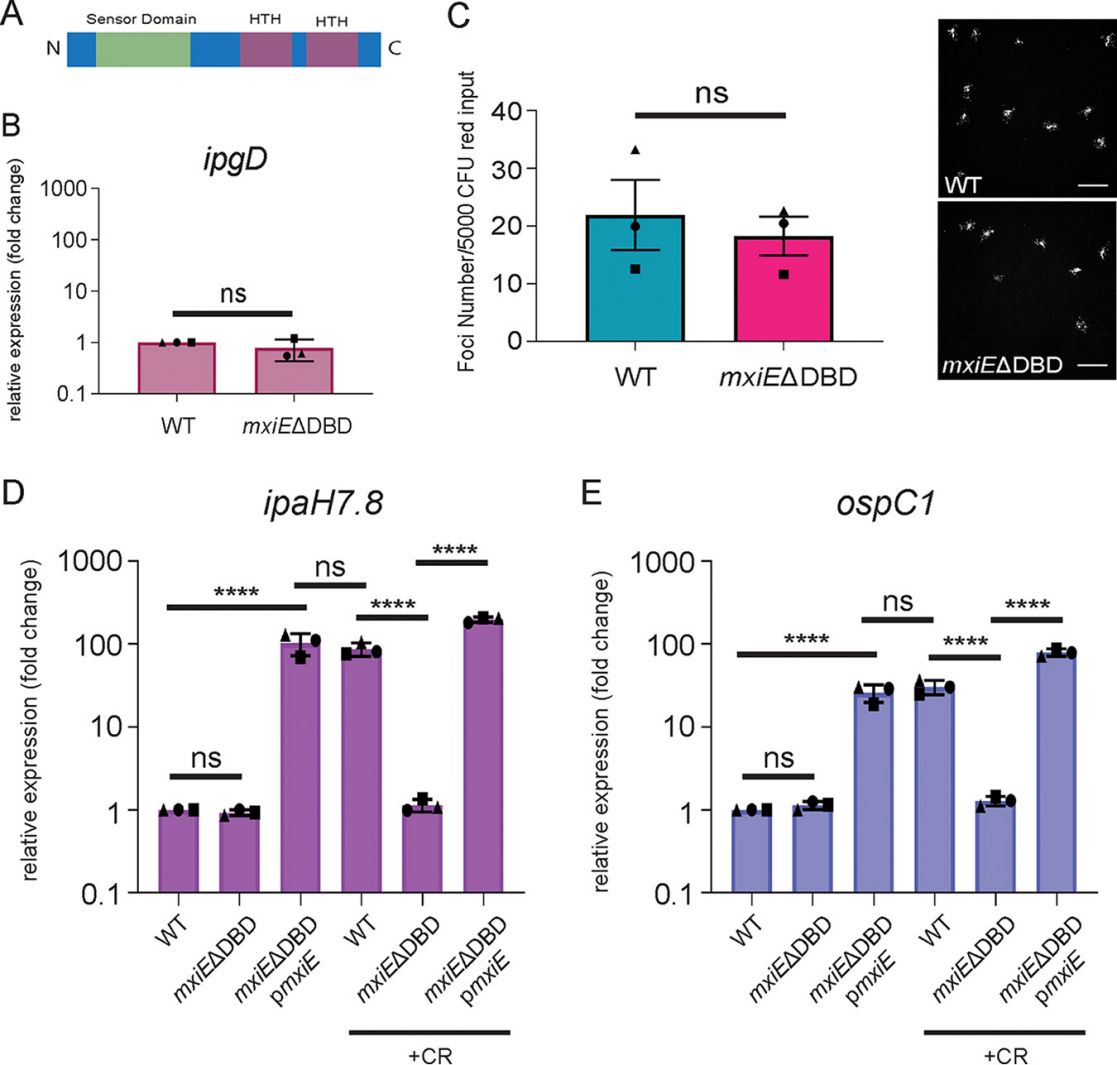
Design and validation of the *mxiE*ΔDBD strain. Schematic of the MxiE protein domains, including the DNA-binding domain (DBD), which is comprised of two helix-turn-helix (HTH) motifs. (B) qPCR of *ipgD* in *mxiE*ΔDBD relative to WT. (C) Invasion assay where the number of foci formed in HT-29 cells was quantified and compared relative to WT. Representative images of infection foci for WT and *mxiE*ΔDBD at 8 h postinfection. The scale bar is 100 μm. WT, *mxiE*ΔDBD, and *mxiE*ΔDBD complemented with pBAD *mxiE* (p*mxiE*) strains were grown at 37°C without and with Congo red dye (CR) and qPCR was performed for (D) *ipaH7.8* and (E) *ospC1* mRNA expression relative to WT without CR. Data shown are the averages of three independent biological experiments and the error bars represent standard deviation. Student’s *t* test (B and C) or a one-way analysis of variance (ANOVA) (D and E) with Tukey’s multiple-comparison test was performed; ns, not significant; ****, *P* < 0.0001.

Previous *mxiE* mutants were generated by the insertion of antibiotic-resistance cassettes (38, 40). Generation of a full *mxiE* deletion mutant was not feasible due to an overlap in reading frames with the downstream gene, *mxiD*, which requires transcriptional slippage for the generation of full-length MxiE to occur (44). To generate a *mxiE* mutant that lacks regulatory function in the 2457T background, we deleted the DNA-binding domain (DBD), which is comprised of 2 helix-turn-helix (HTH) motifs (Fig. 1A, *mxiE*ΔDBD). The Congo red dye (CR) is commonly used to activate S. flexneri type 3 secretion, which leads to the expression of MxiE-dependent genes (45, 46). We used qPCR to assay regulation of MxiE-dependent genes in the *mxiE*ΔDBD mutant compared to wild-type (WT) and found significantly decreased expression of representative MxiE-dependent genes, *ipaH7.8* and *ospC1* (Fig. 1D and E). Importantly, complementation with *mxiE* expressed in *trans* from a pBAD arabinose-inducible promoter fully restored gene expression (Fig. 1D and E). Deletion of the DBD in *mxiE* did not affect T3SS expression, as determined by *ipgD* expression (Fig. 1B). Accordingly, infection of a human colorectal cell line (HT-29) led to a similar number of infection foci, showing that the *mxiE*ΔDBD mutant strain was as invasive as WT bacteria (Fig. 1C). These data demonstrate that the *mxiE*ΔDBD strain has a regulatory defect for MxiE-dependent genes and that the deletion of the DBD does not affect the expression of the T3SS or virulence.

### Defining the MxiE-dependent regulon.

**FIG 2:**
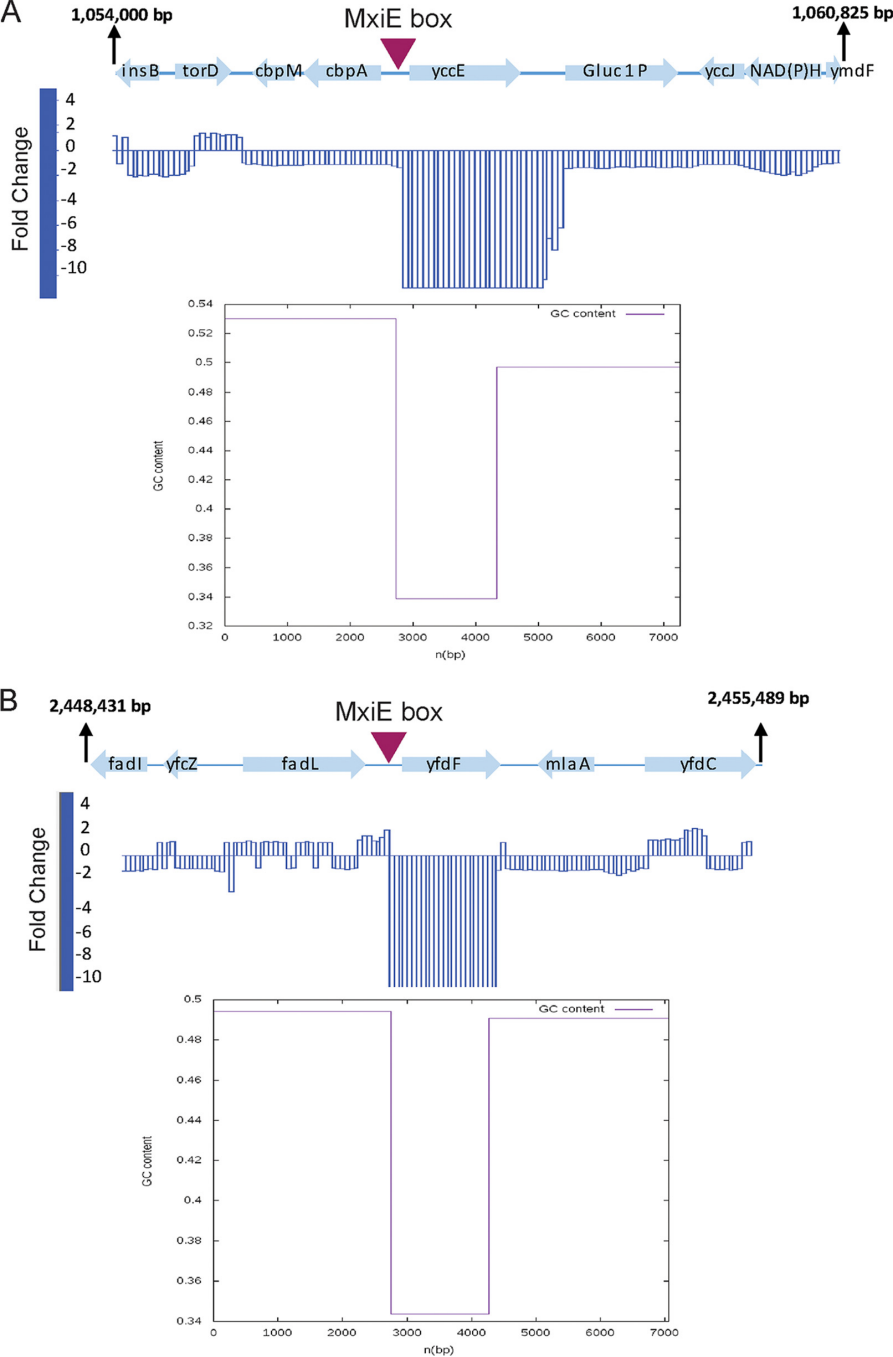
RNA-seq identified MxiE-dependent chromosomal genes. Gene expression peaks from the RNA-seq data comparison of *mxiE*ΔDBD versus wild-type S. flexneri, both with the addition of Congo red dye, which revealed chromosome-encoded genes (A) *yccE* and (B) *yfdF* that were downregulated in the *mxiE*ΔDBD mutant compared to wild-type. MxiE box motifs found using FIMO bioinformatic analysis are indicated with the arrowheads. GC content for (A) *yccE* and (B) *yfdF* and the flanking regions are depicted below the gene expression peaks.

Utilizing comparative analysis of RNA-seq data, we identified 41 genes whose expression was increased at least 2-fold upon the growth of WT bacteria in the presence of the Congo red dye (Table S1). Except for *zraP* and *spy*, all the differentially expressed genes (DEGs) relied on MxiE for full expression in response to the Congo red dye (Table S1). As expected, we identified known MxiE-dependent T3SS effector genes located on the large virulence plasmid, such as *ipaH7.8* and *ospC1* (Table S1 and Fig. S1C). We also identified T3SS effector genes of the IpaH family located on the chromosome, including *ipaH_*1 (pseudogene in 2457T), *ipaH_2*, *ipaH_4*, *ipaH_5*, and *ipaH_7* (Table S1). Interestingly, we identified additional DEGs located on the chromosome, including *yccE* (gene ID 1077473), *yfdF* (gene ID 1026565), and *yjgL* (gene ID 1027047) (pseudogene in 2457T), whose expression relied on MxiE (Fig. 2A and B and Table S1).

10.1128/msphere.00485-22.1 FIG S1 Validation of MxiE box motif search in RNA-seq identified DEGs. Schematic of bioinformatics analysis to identify putative MxiE box motifs and genes downstream. (B) Resulting in MxiE box motifs from FIMO analysis in the virulence plasmid. MxiE box sequences with the same color have identical sequences. (C) Gene expression peaks in the virulence plasmid from RNA-seq comparison of WT without versus with the addition of Congo red dye. MxiE box sequences were identified as described in (A) and marked with the corresponding color in (B). Download FIG S1, TIF file, 2.1 MB. Copyright © 2022 Hall et al. 2022 Hall et al. https://creativecommons.org/licenses/by/4.0/ This content is distributed under the terms of the Creative Commons Attribution 4.0 International license.

10.1128/msphere.00485-22.6 TABLE S1 Differentially expressed genes from RNA-seq of S. flexneri WT and *mxiE*ΔDBD with and without the addition of Congo red dye. Download Table S1, XLSX file, 0.03 MB. Copyright © 2022 Hall et al. 2022 Hall et al. https://creativecommons.org/licenses/by/4.0/ This content is distributed under the terms of the Creative Commons Attribution 4.0 International license.

To identify putative MxiE box sequences for the RNA-seq DEGs, we conducted a bioinformatic analysis using the MEME suite (Fig. S2A). The MxiE motif (GTATCGTTTTTTTAnAG) search resulted in 47 matches in the virulence plasmid (accession no. NC_004851.1) and 496 matches (with up to 4 potential substitutions) in the chromosome sequence (accession no. AE014073.1) (41, 42). We identified potential MxiE box motifs for 18 of the DEGs relying on MxiE for activation (Table S2). These DEGs included virulence plasmid-encoded effector genes (Fig. S1B) as well as chromosome-encoded IpaH family members (Table S2). Interestingly, we found MxiE box motifs upstream of *yccE*, *yfdF*, *and yjgL* (Fig. 2A and B and Table S2). Altogether, these data identify chromosomal genes whose expression is dependent on the activity of the T3SS and MxiE.

10.1128/msphere.00485-22.2 FIG S2 Phylogenetic tree of homologous proteins to YccE. An unrooted phylogenetic tree depicting evolutionary relationships of the identified homologous proteins to YccE using representatives of phylogroups of *Shigella* spp. and E. coli. The phylogroups are indicated in the tree as the first character before the bacterial name as well as with colors (i.e., *Shigella* [red], A [yellow], B1 [pink], C [purple], D [blue], or E [green]). Download FIG S2, TIF file, 1.4 MB. Copyright © 2022 Hall et al. 2022 Hall et al. https://creativecommons.org/licenses/by/4.0/ This content is distributed under the terms of the Creative Commons Attribution 4.0 International license.

10.1128/msphere.00485-22.7 TABLE S2 MxiE box motifs identified upstream of RNA-seq differentially expressed genes. Download Table S2, XLSX file, 0.1 MB. Copyright © 2022 Hall et al. 2022 Hall et al. https://creativecommons.org/licenses/by/4.0/ This content is distributed under the terms of the Creative Commons Attribution 4.0 International license.

### Validation of *yccE* and *yfdF* as MxiE-dependent genes.

**FIG 3:**
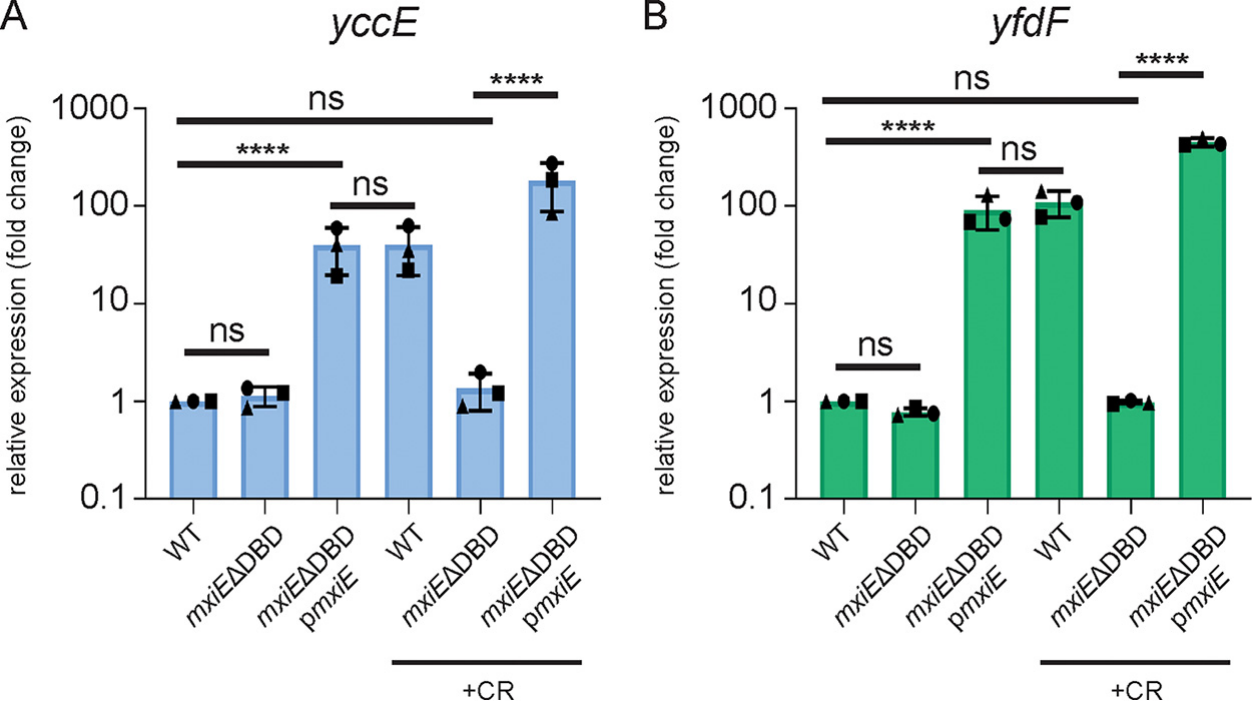
MxiE is necessary for *yccE* and *yfdF* expression. WT, *mxiE*ΔDBD, and *mxiE*ΔDBD complemented with pBAD *mxiE* (p*mxiE*) strains were grown at 37°C without and with Congo red dye (CR) and qPCR was performed for (A) *yccE* and (B) *yfdF* mRNA expression relative to WT. Data shown are the averages of three independent biological experiments and the error bars represent standard deviation. One-way ANOVA with Tukey’s multiple-comparison test was performed; ns, not significant; ****, *P* < 0.0001.

To validate our RNA-seq results, we sought to confirm the necessity of MxiE for the expression of the representative chromosomal genes of interest, *yccE* and *yfdF* (Fig. 3, WT and *mxiE*ΔDBD −/+CR). We did not pursue *yjgL* because it is a pseudogene in S. flexneri 2457T. We performed qPCR using RNA isolated from bacterial cultures of the *mxiE*ΔDBD mutant and WT *Shigella* with overexpression of *mxiE* from an arabinose-inducible pBAD promoter. As expected, we saw a regulatory defect in the *mxiE*ΔDBD mutant. However, when *mxiE* was overexpressed upon the addition of arabinose, we observed a rescue of expression to WT levels (Fig. 3A and B). These data, therefore, confirm our RNA-seq results and demonstrate that MxiE is required for the expression of *yccE* and *yfdF*.

**FIG 4:**
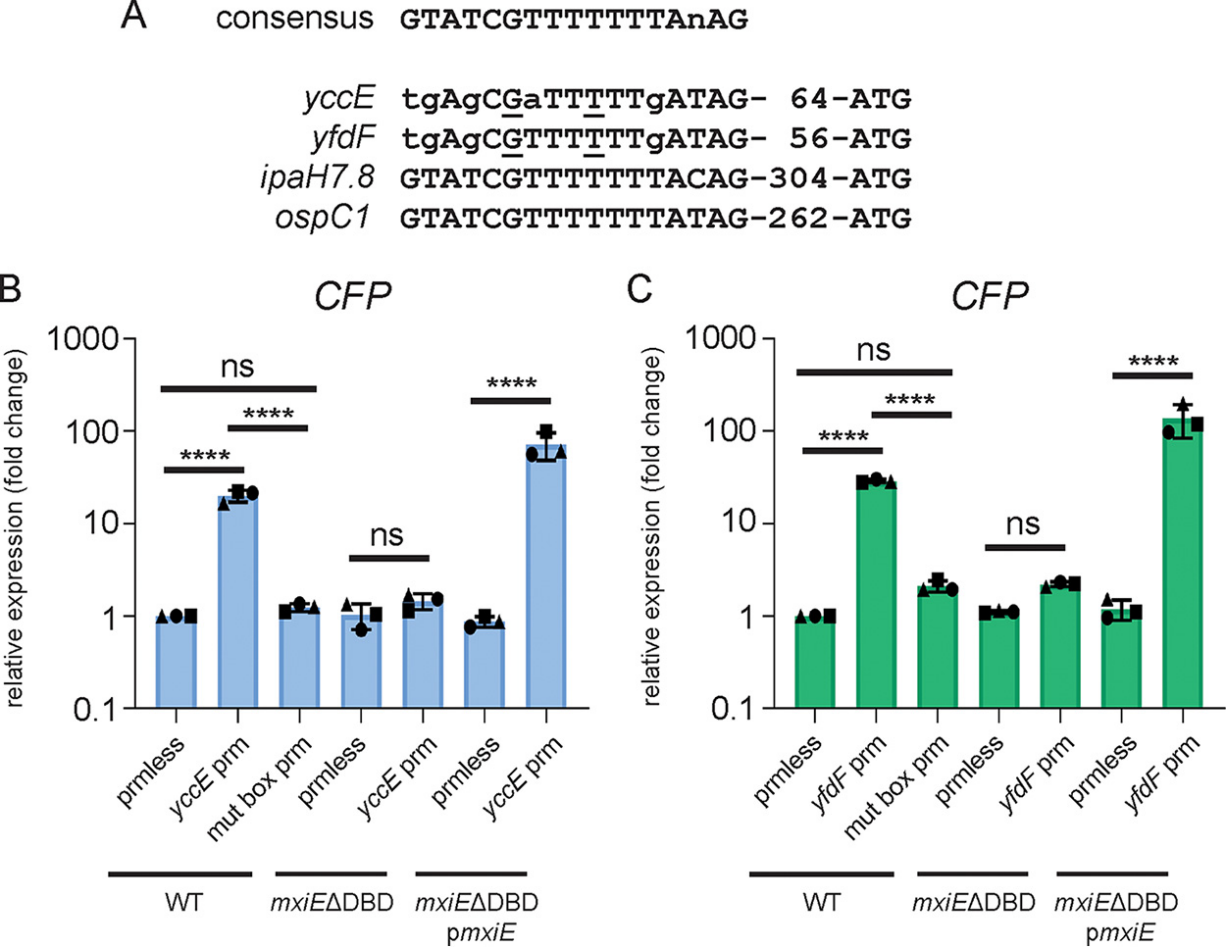
*yccE* and *yfdF* have functional MxiE box *cis*-regulatory elements. Alignment of the MxiE box sequences of MxiE-dependent genes with mutation sites underlined. WT, *mxiE*ΔDBD, and *mxiE*ΔDBD complemented with pBAD *mxiE* (p*mxiE*) strain harboring the CFP reporter constructs, promoter-less control (prmless), *yccE*/*yfdF* promoter (*yccE*/*yfdF* prm), and *yccE*/*yfdF* mutated MxiE box promoter (mut box prm), were grown at 37°C with Congo red dye (CR) to induce T3SS secretion and qPCR was performed for CFP mRNA expression as a proxy for (B) *yccE* and (C) *yfdF* promoter activation relative to WT promoter-less. Data shown are the averages of three independent biological experiments and the error bars represent standard deviation. One-way ANOVA with Tukey’s multiple-comparison test was performed; ns, not significant; ****, *P* < 0.0001.

Our bioinformatic analysis supported the notion that *yccE* and *yfdF* harbor a putative MxiE box in the vicinity of their coding region (Fig. 2 and Table S2). To test the functionality of these putative MxiE box sequences we used a cyan fluorescent protein (CFP) reporter construct with or without mutations (G6>C and T10>A) in the 17-bp MxiE box of *yccE* and *yfdF* and assayed CFP expression as a readout of promoter activation (nucleotides mutated are underlined in Fig. 4A). We found that the WT promoter resulted in significant CFP expression compared to the promoter-less control, and the introduction of the mutations in the MxiE box abolished activation in response to the Congo red dye (Fig. 4B and C). As a control, we assayed promoter activation in the *mxiE*ΔDBD mutant and as expected, we observed no significant activation of the WT *yccE* and *yfdF* promoters. However, activation was rescued by overexpressing *mxiE* using a pBAD arabinose-inducible construct (Fig. 4B and C). These results provide evidence that the chromosomal genes *yccE* and *yfdF* have a functional MxiE box in their promoter regions.

### *yccE* phylogeny analysis.

While virulence plasmid-encoded MxiE-dependent genes are uniquely found in *Shigella* spp., we found that, similar to *yfdF*, the chromosome-encoded gene *yccE* is conserved in E. coli (47) (Fig. S2). The representatives of the phylogroups known to be closely related to S. flexneri strains revealed the homology of the gene with commensal E. coli strains that were usually classified under the phylogroups A and B1. The sequence homology was not conserved across these phylogroups indicating no significant correlation between phylogenicity and virulence (48, 49). The amino acid sequence alignments revealed more conserved sites in the second half of the gene, suggesting the presence of a conserved domain, although there were no domains predicted for YccE with high confidence. The *yccE* gene was absent in S. boydii and members of the B2 phylogroup and present but fragmented in S. dysenteriae and S. sonnei.

We further analyzed the gene arrangement surrounding the *yccE* locus using the Pathosystems Resource Integration Center (PATRIC) database. The E. coli strain MG1655 was the closest match to the gene arrangement at the *yccE* locus in S. flexneri strain 2a 301. In contrast, in addition to reductases, phosphatase, and chaperone coding genes, IS elements (A to D) and genes encoding phage proteins were found interspersed in S. flexneri 2a 301 and S. dysenteriae Sd197 (Fig. S3). Altogether, these data reveal the conservation of the *yccE* gene across many E. coli and *Shigella* strains, with great variability of gene organization in the chromosome region surrounding the *yccE* locus.

10.1128/msphere.00485-22.3 FIG S3 Gene arrangement for regions around *yccE*. Gene arrangement analysis for a 10,000 bp region, including *yccE* for different *Shigella* spp. and nonpathogenic (K-12) and pathogenic (O157:H7) E. coli species. The length and direction of the arrows are indicative of gene length and orientation. The numbers on the arrows indicate the protein or insertion elements: (i) trimethylamine-N-oxide reductase (TorA), (ii) TorA specific chaperone protein TorD, (iii) chaperone modulator protein CbpM, (iv) DNA-J class molecular Chaperone CbpA, (v) YccE, (vi) glucose-1-phosphatase, (vii) YccJ, (viii) NAD(P)H dehydrogenase, (ix) YmdF uncharacterized protein, (x) hypothetical protein, (xi) pyrimidine permease RutG, (xii) FMN reductase, (xiii) InsB, (xiv) InsA, (xv) hypothetical protein, (xvi) hypothetical protein, (xvii) hypothetical protein, (xviii) phage integrase, (xix) phage protein, (xx) phage protein, (xxi) transposase, (xxii) Ins C, and (xxiii) Ins D. Download FIG S3, TIF file, 1.2 MB. Copyright © 2022 Hall et al. 2022 Hall et al. https://creativecommons.org/licenses/by/4.0/ This content is distributed under the terms of the Creative Commons Attribution 4.0 International license.

### H-NS represses *ipaH7.8*, *ospC1*, *yccE*, *yfdF*, and the E. coli homologous genes *yccE* and *yfdF*.

Previous observations suggested that virulence genes encoding the T3SS as well as MxiE-dependent T3SS effector proteins, are AT-rich compared to housekeeping genes in S. flexneri (7). We, thus, conducted a bioinformatic analysis of the GC content at the *yccE* and *yfdF* loci, including 3000 bp flanking their respective coding region (Fig. 2). The analysis of the GC profile segmentation revealed a dramatic AT enrichment (>65%) at both loci compared to the surrounding chromosomal regions (Fig. 2A and B).

**FIG 5:**
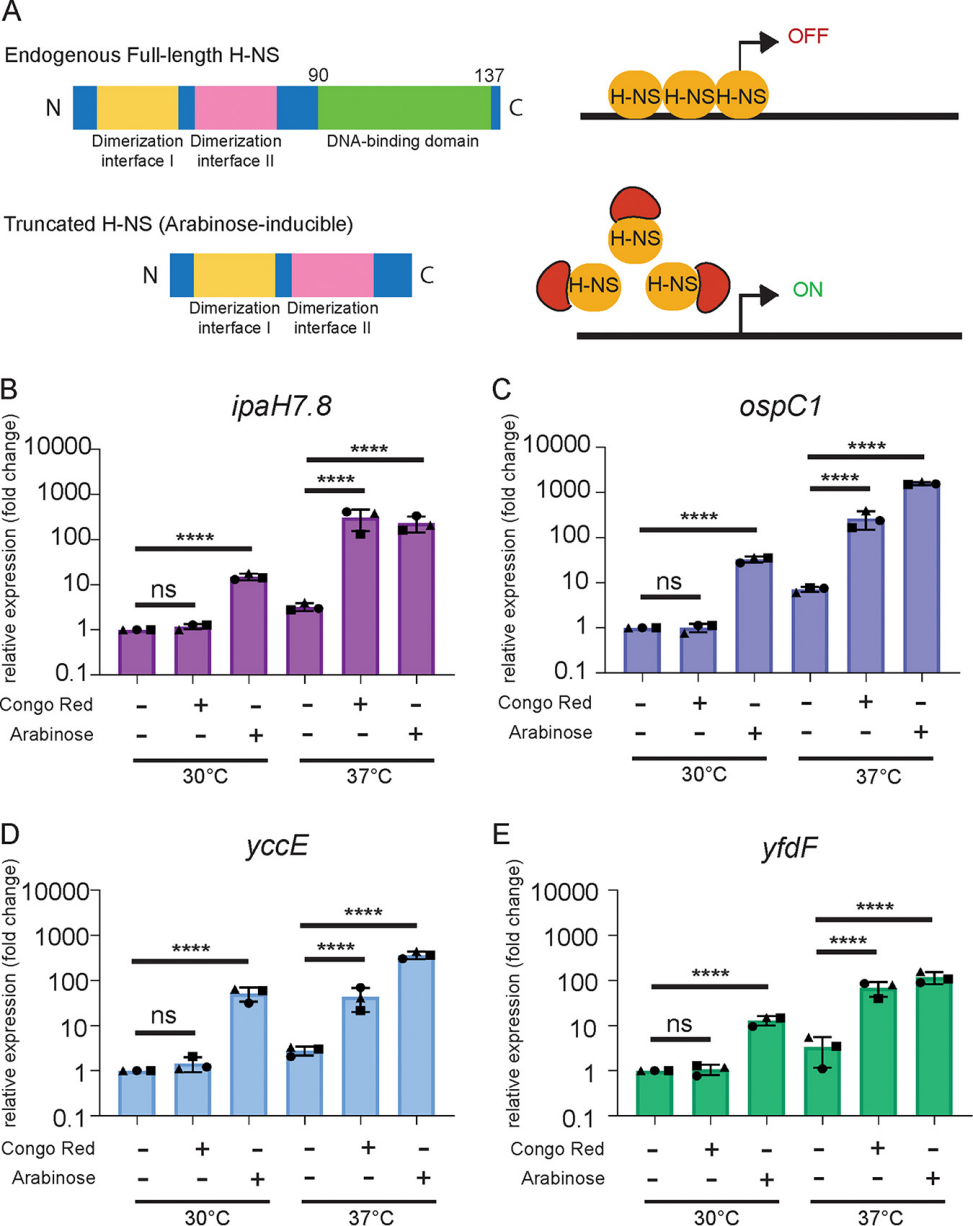
Sequestration of H-NS in S. flexneri leads to *ipaH7.8*, *ospC1*, *yccE*, and *yfdF* expression in nonpermissive conditions. Schematic of the H-NS dominant negative overexpression system where a truncated H-NS (ΔDBD) was induced by arabinose and oligomerizes with endogenous H-NS preventing DNA-binding and transcriptional repression. The S. flexneri WT strain with the dominant negative H-NS construct was grown at 30°C or 37°C with either no arabinose (−Ara), with arabinose (+Ara), or no arabinose but with Congo red (−Ara +CR) and qPCR was performed for mRNA expression levels of (B) *ipaH7.8*, (C) *ospC1*, (D) *yccE*, and (E) *yfdF*. Data shown are the averages of three independent biological experiments and the error bars represent standard deviation. One-way ANOVA with Tukey’s multiple-comparison test was performed; ns, not significant; ***, *P* = 0.0002; ****, *P* < 0.0001.

H-NS, a protein involved in the organization and regulation of the nucleoid, is known to preferentially bind AT-rich regions of DNA (i.e., low GC content) (50, 51). Therefore, we hypothesized that H-NS may be involved in the transcriptional repression of MxiE-dependent genes, including *yccE* and *yfdF*. Failed attempts to generate a full deletion *hns* mutant in S. flexneri 2457T led us to suspect that a strain lacking *hns* may not be viable. We note that existing *hns* mutants were obtained by transposon mutagenesis, which resulted in the production of a truncated polypeptide (10, 17). We, thus, turned to a dominant negative overexpression system, where a truncated form of H-NS is overexpressed using a pBAD arabinose-inducible promoter (p*hns*ΔDBD). Similar truncations of H-NS displaying dominant negative properties have been used in enteropathogenic E. coli (EPEC) and Yersinia enterocolitica (52, 53). By overexpressing the oligomerization domain of H-NS, the endogenous full-length H-NS that is capable of DNA binding is sequestered, allowing for the activation of H-NS-repressed genes (Fig. 5A). We validated the dominant negative system by showing that the H-NS-regulated gene, *virB*, was indeed de-repressed upon overexpression of the dominant negative H-NS, while the expression of the housekeeping gene, *dnaA*, was unaffected (Fig. S4) (17).

10.1128/msphere.00485-22.4 FIG S4 Validation of the dominant negative H-NS overexpression system. The S. flexneri WT strain with the dominant negative H-NS construct was grown at 30°C or 37°C with either no arabinose (−Ara) or with 0.2% arabinose (+Ara) and qPCR was performed for mRNA expression levels of positive control for H-NS repression (A) *virB* and a negative control (B) *dnaA*. Data shown are the averages of three independent biological experiments and the error bars represent standard deviation. One-way ANOVA with Tukey’s multiple-comparison test was performed; ns, not significant; **, *P* < 0.01; ****, *P* < 0.0001. Download FIG S4, TIF file, 0.9 MB. Copyright © 2022 Hall et al. 2022 Hall et al. https://creativecommons.org/licenses/by/4.0/ This content is distributed under the terms of the Creative Commons Attribution 4.0 International license.

Using *ipaH7.8* and *ospC1* as representative virulence plasmid-encoded MxiE-dependent genes, we observed that sequestration of H-NS in WT *S. flexneri* led to a significant increase in MxiE-dependent gene expression, similar to the activation observed in the presence of the Congo red dye (Fig. 5B and C). In addition, we found that the expression of the chromosomal genes *yccE* and *yfdF* was also significantly increased upon overexpression of the dominant negative H-NS (Fig. 5D and E). We finally investigated whether H-NS repression of MxiE-dependent genes was conserved in non-pathogenic E. coli. Upon overexpression of the dominant negative H-NS in E. coli DH5α, we observed a significant increase in mRNA expression of the homologous E. coli genes *yccE* and *yfdF* (Fig. S5A and B). We confirmed these results in strain MC4100 and its isogenic *hns* mutant (Fig. S5C and D).

10.1128/msphere.00485-22.5 FIG S5 The E. coli *yccE* and *yfdF* homologous genes are repressed via H-NS. The E. coli K-12 DH5α strain with the dominant negative H-NS construct (p*hns*ΔDBD) was grown with and without Arabinose at 37°C (A and B) and the MC4100 WT and *hns* strains were grown at 37°C (C and D) and qPCR was performed to obtain mRNA expression levels of E. coli homologous genes to (A and C) *yccE* and (B and D) *yfdF* relative to WT without arabinose (A and B) or WT (C and D). Data shown are the averages of three independent biological experiments and the error bars represent standard deviation. Student’s *t* test was performed; ****, *P* < 0.0001. Download FIG S5, TIF file, 1.1 MB. Copyright © 2022 Hall et al. 2022 Hall et al. https://creativecommons.org/licenses/by/4.0/ This content is distributed under the terms of the Creative Commons Attribution 4.0 International license.

Altogether, these results, both WT indicate that H-NS silences the expression of representative MxiE-dependent genes, including *ipaH7.8* and *ospC1* on the virulence plasmid and *yccE* and *yfdF* on the chromosome.

### MxiE is not necessary for the expression of *ipaH7.8*, *ospC1*, *yccE*, and *yfdF* when H-NS is depleted.

**FIG 6:**
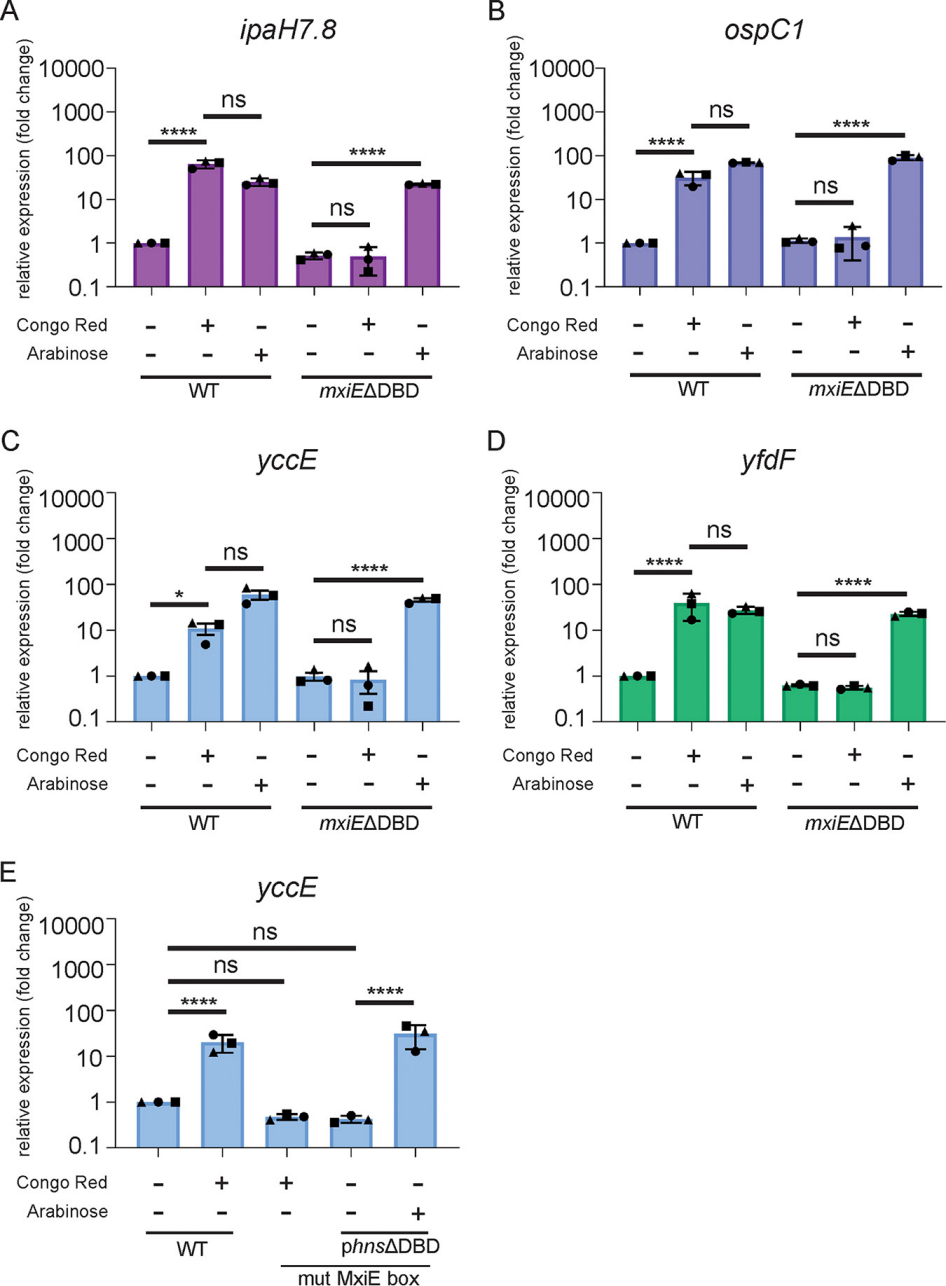
MxiE is not necessary for the expression of *ipaH7.8*, *ospC1*, *yccE*, or *yfdF* when H-NS is depleted. The S. flexneri WT and *mxiE*ΔDBD strains with the dominant negative H-NS construct (p*hns*ΔDBD) were grown at 37°C with or without Congo red and/or Arabinose to induce expression of p*hns*ΔDBD and qPCR was performed for mRNA expression levels of (A) *ipaH7.8*, (B) *ospC1*, (C) *yccE*, and (D) *yfdF* relative to WT. An S. flexneri strain with a mutated MxiE box upstream of *yccE* without and with the dominant negative H-NS construct was grown at 37°C with or without Congo red and/or Arabinose to induce expression of p*hns*ΔDBD and qPCR was performed for the mRNA expression level of (E) *yccE* compared to WT. Data shown are the averages of three independent biological experiments and the error bars represent standard deviation. One-way ANOVA with Tukey’s multiple-comparison test was performed; ns, not significant; **, *P* = 0.008 (ospC1); *P* = 0.0013 (SF1005); ***, *P* = 0.0003; ****, *P* < 0.0001.

Our results obtained in E. coli MC4100, WT and isogenic *hns* mutant strains, led us to hypothesize that MxiE-dependent genes could be fully expressed in the absence of MxiE when H-NS was depleted (Fig. S5C and D). To test this hypothesis in S. flexneri, we compared the expression levels of MxiE-dependent genes in WT and *mxiE*ΔDBD mutant strains overexpressing the H-NS dominant-negative construct. We found that both strains had similar expression levels of *ipaH7.8*, *ospC1*, *yccE*, and *yfdF* (Fig. 6A to D), showing that MxiE was no longer required for their expression when H-NS was depleted.

In a complementary approach, we determined whether the expression of MxiE-dependent genes relies on the MxiE box when H-NS was depleted. To this end, we generated a S. flexneri strain harboring mutations in the MxiE box of *yccE* and introduced the dominant negative H-NS construct in the corresponding strain. As expected, *yccE* activation in response to the Congo red dye was abolished when the MxiE box was mutated (Fig. 6E). However, overexpression of the dominant negative H-NS led to strong activation of *yccE* expression, regardless of the presence of a functional MxiE box (Fig. 6E). Altogether, these results indicate that MxiE is not required for the expression of representative virulence plasmid-encoded genes, such as *ipaH7.8* and *ospC1*, and chromosome-encoded genes, such as *yccE* and *yfdF*, when H-NS is depleted.

### VirB is not required for the expression of *ipaH7.8* and *yccE* upon MxiE/IpgC overexpression.

**FIG 7:**
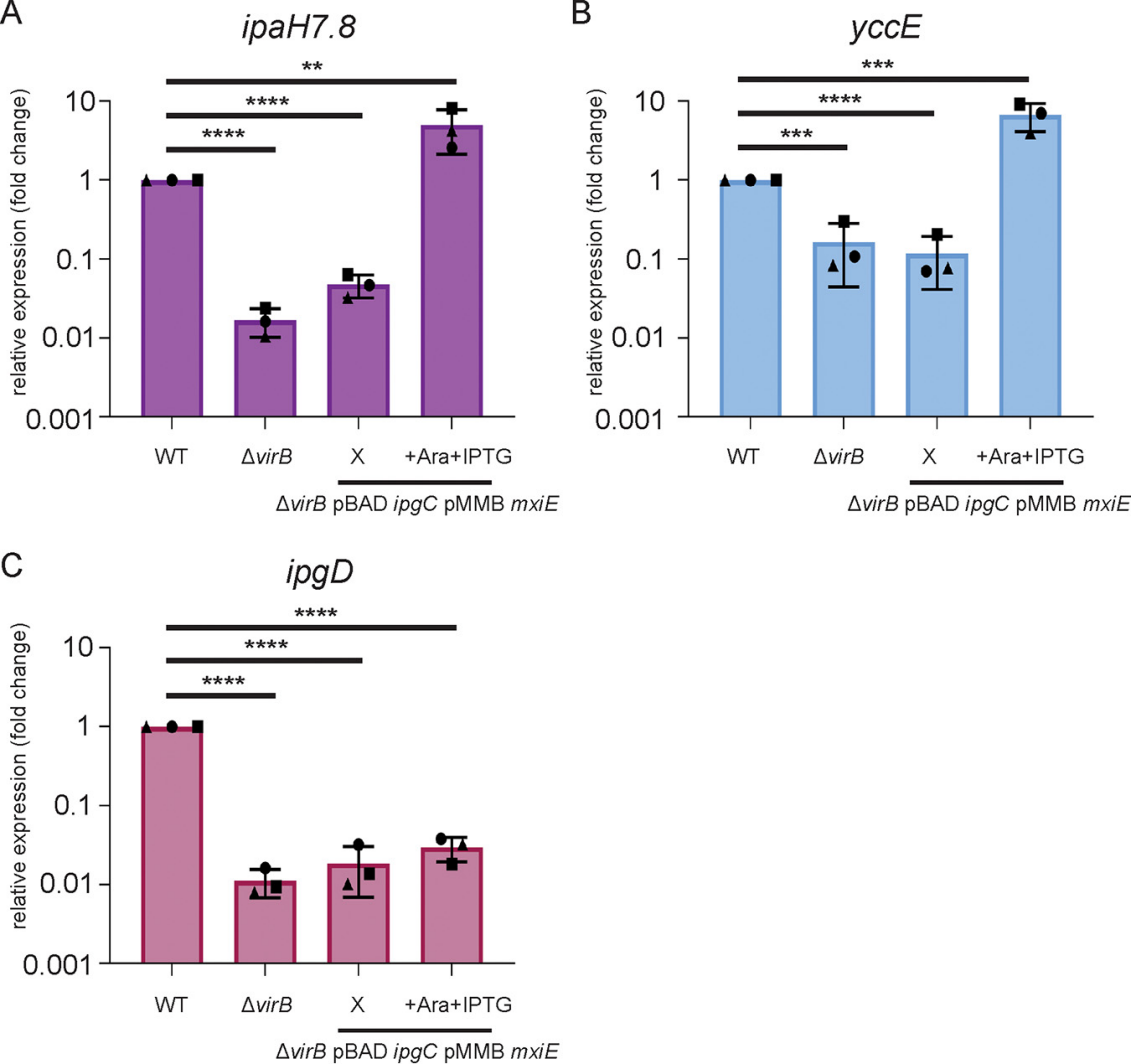
VirB is not required for the expression of *ipaH7.8* or *yccE* upon MxiE/IpgC overexpression. The S. flexneri WT, Δ*virB* deletion strain, and Δ*virB* with pMMB *mxiE* and pBAD *ipgC* were grown at 37°C with Congo red and with either no induction (X) or induction of both *ipgC* and *mxiE* (+Ara +IPTG) and qPCR was performed for mRNA expression levels of (A) *ipaH7.8*, (B) *yccE*, and (C) *ipgD* relative to WT. Data shown are the averages of three independent biological experiments and the error bars represent standard deviation. One-way ANOVA with Tukey’s multiple-comparison test was performed; ns, not significant; **, *P* = 0.0013; ***, *P* = 0.0001 (Δ*virB*); ***, *P* = 0.0002 (+Ara+IPTG); ****, *P* < 0.0001.

To our knowledge, in S. flexneri, VirB is the main protein involved in counteracting the H-NS-mediated silencing of virulence genes (15, 26). To investigate whether VirB may be involved in the expression of MxiE-dependent genes, we first designed a dual expression system in which *mxiE* and *ipgC* are expressed from an Isopropyl β-D-1-thiogalactopyranoside (IPTG)-inducible and arabinose-inducible promoter, respectively. This was necessary because the expression of *mxiE* and *ipgC* depends on VirB (29, 30). Using this system, we determined mRNA expression levels of representative virulence plasmid- and chromosome-encoded MxiE-dependent genes (*ipaH7.8*, *yccE*) in the *virB* mutant strain with and without overexpression of *mxiE* and *ipgC* (Fig. 7A and B). As expected, we observed a significant decrease in *ipaH7.8* and *yccE* expression in the *virB* mutant, due to the dependency of *mxiE* and *ipgC* expression on *virB* expression. Overexpression of *mxiE* and *ipgC* was sufficient to restore mRNA expression of *ipaH7.8* and *yccE* to WT levels in the presence of the Congo red dye (Fig. 7A and B). Importantly, overexpression of *mxiE* and *ipgC* did not restore the expression of *ipgD*, a VirB-dependent gene (Fig. 7C).

Altogether, these results indicate that VirB is not required for the expression of representative MxiE-dependent genes from the virulence plasmid (*ipaH7.8*) and chromosome (*yccE*), upon MxiE/IpgC overexpression.

## DISCUSSION

The MxiE-dependent genes have been determined previously by (i) comparing luciferase reporters for virulence plasmid-encoded genes in wild-type S. flexneri and an *ipaB* constitutive secretion mutant strain, (ii) comparing GFP reporters in the presence or absence of MxiE, and (iii) comparing the transcriptional profiles of wild-type S. flexneri and an *ipaB mxiE* mutant using a macroarray (12, 38, 40). Additionally, a recent study used RNA-seq in a constitutively secreting mutant (*ipaD*) to determine genes, located on the virulence plasmid or on the chromosome, that are differentially expressed based on the activity of the T3SS (43). Here, we used RNA-seq to identify genes that were differentially expressed in response to type 3 secretion induced by the Congo red dye, and we determined whether these genes rely on the integrity of MxiE for full expression (Fig. 3).

Our RNA-seq analysis led to the identification of 41 genes differentially expressed by the Congo red dye (Table S1). Among those, 17 genes were previously shown to be MxiE-dependent, including *ipaH7.8* and *ospC1*. Two chromosome-encoded genes (*yfdF* and *yjgL*) were also discovered in a recent RNA-seq study and were first referred to as *gem*1 and *gem*3 and then renamed as *icaT* and *icaR*, respectively (43, 47) (Fig. 2B and Table S1). In addition to these two genes, we found a third chromosome-encoded gene (*yccE*) that was not previously identified (Fig. 2A). Our bioinformatic analysis revealed the presence of putative MxiE box sequences in the vicinity of all previously identified genes (Table S2). Among the newly identified genes, 3 displayed a putative MxiE box, and we experimentally validated the importance of this regulatory motif for *yccE* and *yfdF* expression (Fig. 4). The apparent absence of a MxiE box in the vicinity of 17 newly identified genes may suggest indirect effects of MxiE on global gene regulation (Table S1 and S2). We also identified two genes (*zraP* and *spy*) whose activation in the presence of the Congo red dye did not rely on MxiE. Interestingly, these two genes may function in the envelope stress response, which may reflect a toxic effect of the Congo red dye on the cell wall or the membrane.

Similar to a previous study conducted on *yfdF*, we found that the chromosome-encoded MxiE-dependent gene *yccE* is conserved in non-pathogenic E. coli strains, such as K-12 (Fig. S2) (47). Additionally, we did not find *yccE* to be highly conserved in other *Shigella* spp. besides S. flexneri. It was absent in S. boydii and S. dysenteriae and fragmented in S. sonnei, and it was also absent from the B2 phylogroup of E. coli (Fig. S2). This agrees with the notion that S. flexneri may have diverged from a different lineage of E. coli than S. sonnei, S. boydii, or S. dysenteriae, as previously suggested (49, 54).

Based on our bioinformatic analysis, we found that the DNA content of *yccE* and *yfdF* is AT-rich compared to the surrounding chromosomal regions (Fig. 2). Given that H-NS preferentially binds to AT-rich regions of DNA, these data, together with the previous analysis of the GC content of the virulence plasmid sequence, led us to hypothesize that H-NS may silence the expression of MxiE-dependent genes (7, 50, 51). Our genetic study using a dominant negative version of H-NS revealed that representative virulence plasmid genes (*ipaH7.8* and *ospC1*) and chromosome-encoded genes (*yccE* and *yfdF*) are repressed by H-NS (Fig. 5). H-NS has been proposed to silence extensive regions of AT-rich loci by first binding to high-affinity nucleation sites (tCGATAAATT) and then spreading along DNA (55). Due to the AT-rich nature of the MxiE box and its similarity with the consensus H-NS binding site, H-NS may use the MxiE box sequence as a portal of entry for nucleation. However, based on our results, where we mutated the MxiE box upstream of *yccE* and did not observe activation unless we overexpressed the dominant negative H-NS (Fig. 6E), it is unlikely that H-NS is using the MxiE box as a unique site for nucleation. Although further mutational analysis of the MxiE box will be required to confirm this assumption, we speculate that multiple high-affinity H-NS binding sites may be present in the AT-rich coding regions of MxiE-dependent genes. In addition to high-affinity binding sites, other factors such as DNA curvature may influence H-NS nucleation along DNA (55). Thus, further experiments are needed to determine how H-NS interacts with DNA at MxiE-dependent loci.

In addition to H-NS, S. flexneri possesses H-NS paralogues, including StpA and Sfh, which can be functionally redundant and form heterodimers with one another (56). The dominant negative H-NS system used in our study is a useful tool for bacterial species in which *hns* deletion is seemingly lethal, such as in enteropathogenic E. coli (EPEC) and Yersinia enterocolitica (52, 53). However, it is important to consider that oligomerization and sequestration can occur with the H-NS-like paralogues as well. While we provide genetic evidence that H-NS is specifically involved in the repression of *yccE* and *yfdF* based on the increased expression of *yccE* and *yfdF* in an E. coli *hns* mutant compared to wild-type (Fig. S5), the potential role of H-NS paralogues in MxiE-dependent gene silencing remains to be investigated.

How does MxiE regulate the expression of MxiE-dependent genes? MxiE is an AraC family member and is generally thought to function as a classical transcriptional activator that binds to the MxiE box overlapping with the −35 region of MxiE-dependent promoters (31, 41). Unlike the vast majority of AraC family members, MxiE-dependent activation relies on the presence of its coactivator, the IpgC chaperone (39, 40). This is similar to the situation in Salmonella enterica serovar Typhimurium, where the AraC family member, InvF, homologous to MxiE, forms a complex with SicA, homologous to IpgC, and activates the expression of InvF-dependent genes, such as *sopB* (57, 58). *sopB* expression is also repressed by H-NS, but InvF is required for *sopB* expression, even in the absence of H-NS (59). This is in contrast with LuxR, a transcriptional regulator of quorum sensing genes in Vibrio harveyi, that is also thought to bind a specific motif and function as a transcriptional activator (60). However, a study by Chaparian et al. (61) demonstrated that the expression of quorum-sensing genes is repressed by H-NS in V. harveyi and that LuxR competes with H-NS for binding at promoter regions, leading to the eviction of H-NS and activation of LuxR-dependent genes. Similarly, ToxR in Vibrio cholerae has also been shown to antagonize H-NS and is no longer required for ToxR-dependent gene expression when *hns* is absent (62). In this context, we found that MxiE is no longer required for *ipaH7.8*, *ospC1*, *yccE*, and *yfdF* expression when H-NS is depleted (Fig. 6). These genetic data support the notion, that similar to LuxR and ToxR, MxiE may function to counter-silence H-NS. Additional biochemical studies will be required to further explore this notion.

Previous work established the existence of two mechanisms of H-NS anti-silencing in S. flexneri. One mechanism, occurring at the *virF* promoter, involves changes in DNA topology upon a temperature shift to 37°C, resulting in H-NS release (11, 19, 20). The other mechanism is mediated by VirB, which binds to regulatory motifs located in the promoter regions of *icsB*, *icsP*, *ospD1*, and *ospZ* and is thought to subsequently dislodge H-NS via oligomerization along DNA (11, 13, 23, 26–28, 63). Importantly, our work demonstrates that MxiE/IpgC overexpression at 37°C is sufficient for the expression of *ipaH7.8* and *yccE* in a *virB* deletion strain, showing that VirB is not directly required for the antisilencing of these MxiE-dependent genes under these experimental conditions (Fig. 7A and B).

**FIG 8:**
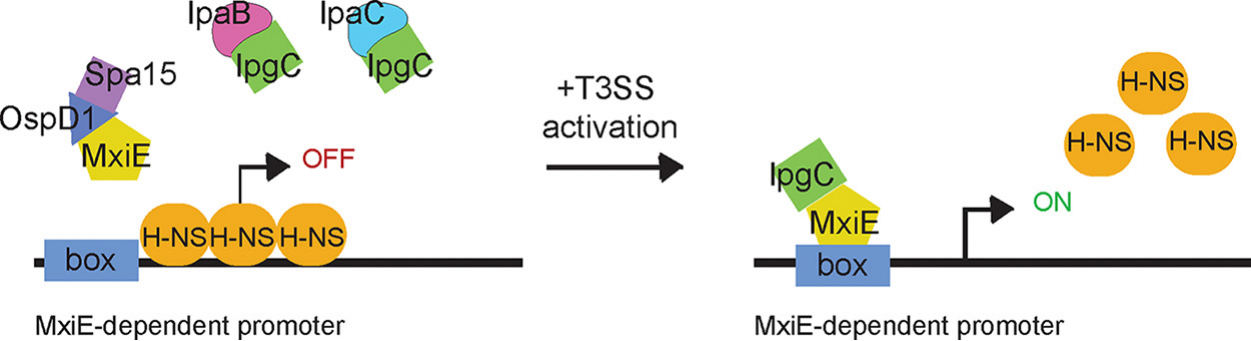
Proposed model of MxiE- and H-NS-dependent regulation in S. flexneri. Before T3SS activation at 37°C, MxiE is bound by the antiactivator, OspD1, whose chaperone is Spa15, and IpgC is functioning as a chaperone for the translocon proteins, IpaB and IpaC. H-NS is repressing the transcription of MxiE-dependent genes. Upon T3SS activation, IpaB and IpaC form the translocon pore and OspD1 is secreted. Transcription of MxiE-dependent genes is activated when MxiE and IpgC are both free to interact and MxiE binds to the box sequence upstream, which results in the counter-silencing of H-NS.

In summary, our genetic study provides support for a model in which MxiE mediates MxiE-dependent gene expression by counteracting H-NS-mediated silencing (depicted in Fig. 8).

## MATERIALS AND METHODS

### Invasion assay.

Human cancer-derived colonic epithelial cells, HT-29 (ATCC HTB-38), and stably expressing plasma membrane-targeted YFP (mbYFP) were used for *Shigella* invasion assays (64). HT-29 mbYFP cells were cultured at 37°C and 5% CO_2_ in McCoy’s 5A medium (Gibco) that was supplemented with 10% heat-inactivated fetal bovine serum (FBS) (Invitrogen). Invasion assays were performed in 96-well assay plates (Corning, catalog number 3904) with confluent HT-29 mbYFP monolayers. Briefly, McCoy’s medium containing exponential-phase *Shigella* containing a pMMB207 plasmid expressing CFP (pCFP) under an IPTG-inducible promoter was added to each well. The 96-well plate was then centrifuged at 1,000 rpm for 5 min to bring the bacteria into contact with the cell monolayer. The infection plate was then incubated at 37°C for 1 h before adding gentamicin (50 μg/mL) to kill extracellular bacteria and IPTG (10 mM) to induce expression of pCFP in the bacteria. At 8 h postinfection, the medium in each well was aspirated and replaced with 4% paraformaldehyde/PBS to fix for 20 min at room temperature. After fixation, wells were washed 3× with 1× PBS before imaging. Each well was imaged using an ImageXpress Micro imaging system (Molecular Devices). The average number of infection foci per well was calculated using the CFP channel and normalized to the bacterial Congo red-positive CFU input. Three independent biological replicates were performed.

### Bacterial strains and growth conditions.

The *Shigella* wild-type strain used in this study was strain 2457T (65). Mutant strains (*mxiE*ΔDBD, Δ*virB*, and *yccE* mutated MxiE box) were generated using a suicide vector, pSB890, for allelic exchange and homologous recombination (66). E. coli strains SM10λpir and Δ*nic*35 were used during the generation of mutant *Shigella* strains for the maintenance of pSB890 constructs and conjugation, respectively. E. coli strain DH5α was used for cloning purposes. E. coli K-12 MC4100, both the WT strain and the *hns* mutant, were kindly provided by Marcia Goldberg (67, 68).

Before liquid culture, *Shigella* and E. coli strains were streaked to isolation from frozen glycerol stocks on LB (lysogeny broth, Fisher) agar plates containing the appropriate antibiotic for selection and incubated at 37°C overnight. Using a single isolated colony, overnight cultures in LB medium were grown rotating on a wheel at 30°C or 37°C, for *Shigella* and E. coli strains, respectively.

For gene expression experiments, *Shigella* and E. coli cultures were grown at 30°C or 37°C (as indicated) on a rotating wheel for either 3 h or 6 h (dominant negative H-NS experiments) after back dilution (1:100) of overnight culture in 5 mL LB medium. Congo red dye (Fisher) was added (100 μg/mL) to LB agar to select red, i.e., virulence plasmid-containing, colonies and at the time of back dilution of liquid *Shigella* cultures to activate secretion, i.e., MxiE-dependent gene activation. l-arabinose (0.2%, p*hns*ΔDBD, and p*ipes*; 1%, p*mxiE*) (Sigma) and isopropyl-β-d-thiogalactopyranoside (IPTG) (1 mM, p*mxiE*) (Fisher) were added at time of back dilution to induce expression of pBAD and pMMB constructs, respectively. 2,6-diaminopimelic acid (DAP) (Sigma Life Sciences) was supplemented (100 μg/mL) to LB medium for the growth of E. coli strain Δ*nic*35. Depending on the strain and/or plasmid antibiotic resistance, the following antibiotics were supplemented in LB medium and agar: ampicillin (100 μg/mL), chloramphenicol (10 μg/mL), tetracycline (10 μg/mL), spectinomycin (100 μg/mL), and kanamycin (30 μg/mL).

### Plasmids and cloning.

All primers (with restriction sites for cloning indicated) used in this study are listed in Table S3. Overexpression constructs were made using either an arabinose-inducible pBAD promoter in vector pBAD18 (ATCC 87393) or an IPTG-inducible promoter in vector pMMB207 (ATCC 37809). The CFP-reporter constructs were made by cloning the promoter of interest upstream of CFP in a pMMB207 vector backbone. Briefly, we introduced mCherry, under the IPTG-inducible promoter, into pMMB207 with a linker and subsequently cloned in CFP with upstream KpnI/BglII cut sites for introducing promoter regions of interest (this study, Table S3). The promoter-less control was the pMMB CFP reporter without a promoter introduced upstream. All plasmid constructs were verified using Sanger sequencing.

10.1128/msphere.00485-22.8 TABLE S3 List of primers used in this study. Download Table S3, XLSX file, 0.02 MB. Copyright © 2022 Hall et al. 2022 Hall et al. https://creativecommons.org/licenses/by/4.0/ This content is distributed under the terms of the Creative Commons Attribution 4.0 International license.

Plasmid DNA was isolated using a Miniprep kit (Qiagen). Ligations were performed using digested plasmid and PCR product DNA with T4 DNA ligase (New England Biolabs). Ligations were transformed into chemically competent DH5α or SM10λpir cells by heat shock at 42°C for 1 min, followed by the addition of Super Optimal broth with Catabolite repression (SOC) medium, recovery at 37°C for 1 h, and subsequent plating onto LB agar with the appropriate antibiotic. pSB890 constructs were transformed into electrocompetent Δ*nic*35 cells by electroporation using a MicroPulser (Bio-Rad) with the Ec2 setting, followed by recovery in SOC medium for 1 h at 37°C on a rotating wheel and subsequent plating on LB agar-supplemented with DAP and the appropriate antibiotic. pBAD and pMMB constructs were transformed into *Shigella* strains following multiple sterile water washes of a 10 mL exponential-phase bacterial pellet followed by resuspension in 100 μL of sterile water with 10% glycerol added to generate competent bacteria. Plasmid DNA (~100 to 400 ng) was added to the tube of the competent *Shigella* and incubated on ice for 15 min. Following incubation with the DNA, the *Shigella* was electroporated and subsequently recovered and plated as detailed above.

### RNA extraction, cDNA synthesis, and qPCR.

The pelleted bacteria were resuspended in 1 mL of TRIzol reagent (Fisher). RNA was separated via chloroform extraction by adding 200 μL of chloroform to the TRIzol resuspension and vortexing to mix. The layers were separated by centrifugation at 12,000 rpm for 15 min at 4°C. The aqueous layer was transferred to a new tube and subsequently used in the Ribopure Bacterial RNA extraction kit (Invitrogen). Following RNA elution of the column, RNA was DNase I-treated using the kit reagents for 30 min at 37°C. DNase I-treated RNA was used to synthesize cDNA using SuperScript II reverse transcriptase and random primers (Invitrogen). Synthesized cDNA was diluted 1:5 with nuclease-free water before use.

qPCR was performed using a LightCycler 96 (Roche) with either the probe-based method (Roche Universal Probe Library) or SYBR green (Bio-Rad), depending on assay design availability. Primers used for qPCR are listed in Table S3. The ΔΔC_t_ method of analysis was performed to determine the relative fold change in gene expression compared to a control group. The housekeeping gene *rpoB* was used for normalization.

### RNA-seq, library construction, and sequencing.

RNA from WT and *mxiE*ΔDBD mutant S. flexneri cultures, with and without Congo red supplementation, was extracted as described above. Library preparation and RNA sequencing were outsourced to Novogene (CA, USA). Briefly, the library construction included steps of total RNA qualification, mRNA enrichment using Ribo-Zero rRNA removal kit specific for bacteria rRNA (Illumina, number MRZMB126), cDNA synthesis, end repair and adaptor ligation, size selection of fragments, and PCR followed by quality check. The quality checks before and after the library construction were performed using Nanodrop and agarose gel electrophoresis, including an integrity check on a 2100 Bioanalyzer (Agilent Technologies) before sequencing. The NEB Next Ultra II RNA Library Prep kit for Illumina (BioLabs, New England, MA, USA) was used for sequencing starting with the step of strand-specific library synthesis where the dTTPs were replaced by dUTPs during the synthesis of the second strand cDNA. The overhangs were converted to blunt ends, followed by adenylation of 3′ ends and adapter ligation using RNA 5′ and RNA 3′ adapters included in the kit. A library concentration of 1.5 ng/μL was used after ensuring the inset size in a quality assessment before sequencing. The resulting libraries were sequenced on Illumina where the libraries constructed were further subjected to cluster growth and sequencing, image acquisition, and base calling steps.

### RNA-seq data analysis.

The raw reads from the sequencing were subjected to analysis of adapter read identification and trimming of the same using Trimmomatic (69). Quality check pre and postadapter trimming were performed using FastQC (70). Both alignment-based and alignment-independent methods were used to align the clean paired-end reads generated for the four samples. The genome sequence available in NCBI (chromosome, accession no. AE014073.1; plasmid pCP301, accession no. NC_004851.1) was used for alignment-based methods using Bowtie for Illumina (71). Fold change calculations were performed from the counts generated using featureCounts where annotations for gene regions were provided in the GTF format and transcripts per million (TPM) values using Kallisto (72, 73). The pseudo alignment was performed using a reference transcriptome file for Kallisto and TPM values were generated. The counts were normalized considering the variation in size of data sets, various gene lengths, and gene assignments for estimated counts less than 100 in either one of the samples being compared were eliminated. The resulting values were used to calculate fold change and log_2_ change values. The upregulated genes were tabulated based on these calculations (Table S1). RNA-seq results were validated via qPCR with three biological replicates for the genes of interest, such as *yccE* and *yfdF*.

### MxiE motif search.

The chromosome and plasmid sequence used for reference-based alignment (chromosome: AE014073.1 and plasmid pCP301: NC_004851.1) was subjected to motif search using FIMO (74). An xml file was prepared by tabulating scores for the four nucleotides encoding the known MxiE box sequence (41, 42, 75). This xml file was used to scan the chromosomal and plasmid sequence individually and the motif sequence matches were retrieved. Based on the location homology with the genes enlisted in Table S2 (upregulated and downregulated) the motif matches were retrieved. All the motif matches falling within the 400 bp flanking regions of the MxiE-dependent genes in chromosome and plasmid sequence were retrieved. In the case of known motifs that exceeded the length from the genes,>400bp the sequences were manually aligned. The identified genes displaying the motif sequence were tabulated (Table S2).

Bamcompare from deepTools2 was used to compare the reads from RNA-Seq MxiEwCR and WTwCR aligned to the reference chromosome accession no. AE014073.1 and plasmid pCP301 accession no. NC_004851.1 sequence in Galaxy server (76) (https://galaxyproject.org/). Here, the genome was divided into bins of 50 bp size, and the overlapping reads were used to calculate differences by subtracting the input from counts measured in the experimental (MxiEwCR) data set. In the case of partial overlaps, the respective fraction was considered in the calculations. A binary BigWig file was generated for visualization in IGB in alignment with the plasmid sequence and its gtf annotation file (77). The peak regions were visualized, and the virulence genes encoded within the peak regions were identified through alignments with the plasmid sequence (221,618 bp). The advanced search option in IGB was used to view the motifs which were color-coded based on similarity in motif sequences and their location could be mapped based on sequence searches and annotation files.

### GC percentage.

The GC percentage of regions of interest was calculated using the GC-Profile tool with suggested parameters halting parameter set to 10, minimum length to segment set to 100 bp, and gap less than 10% in the input sequences were filtered (78).

### *yccE* phylogeny analysis.

The 1257 bp sequence *yccE* gene sequence was analyzed for phylogenetic relationships using the neighbor-joining and Jones-Taylor-Thornton (JTT) matrix-based method for a bootstrap consensus tree with distances in MEGA11 and edited in iTOL (79, 80). Representatives of previously defined phylogroups were selected.

### Data availability.

The RNA-seq data sets generated in this study will be available under the BioProject number PRJNA868769 and SRA number SRR21059953 to SRR21059956.
