# Supplementary figures and images for: Characterization of MxiE- and H-NS-Dependent Expression of ipaH7.8, ospC1, yccE, and yfdF in Shigella flexneri
Source: mSphere. 2022 Nov 8;7(6):e00485-22. doi: 10.1128/msphere.00485-22 (PMC9769918; doi:10.1128/msphere.00485-22)

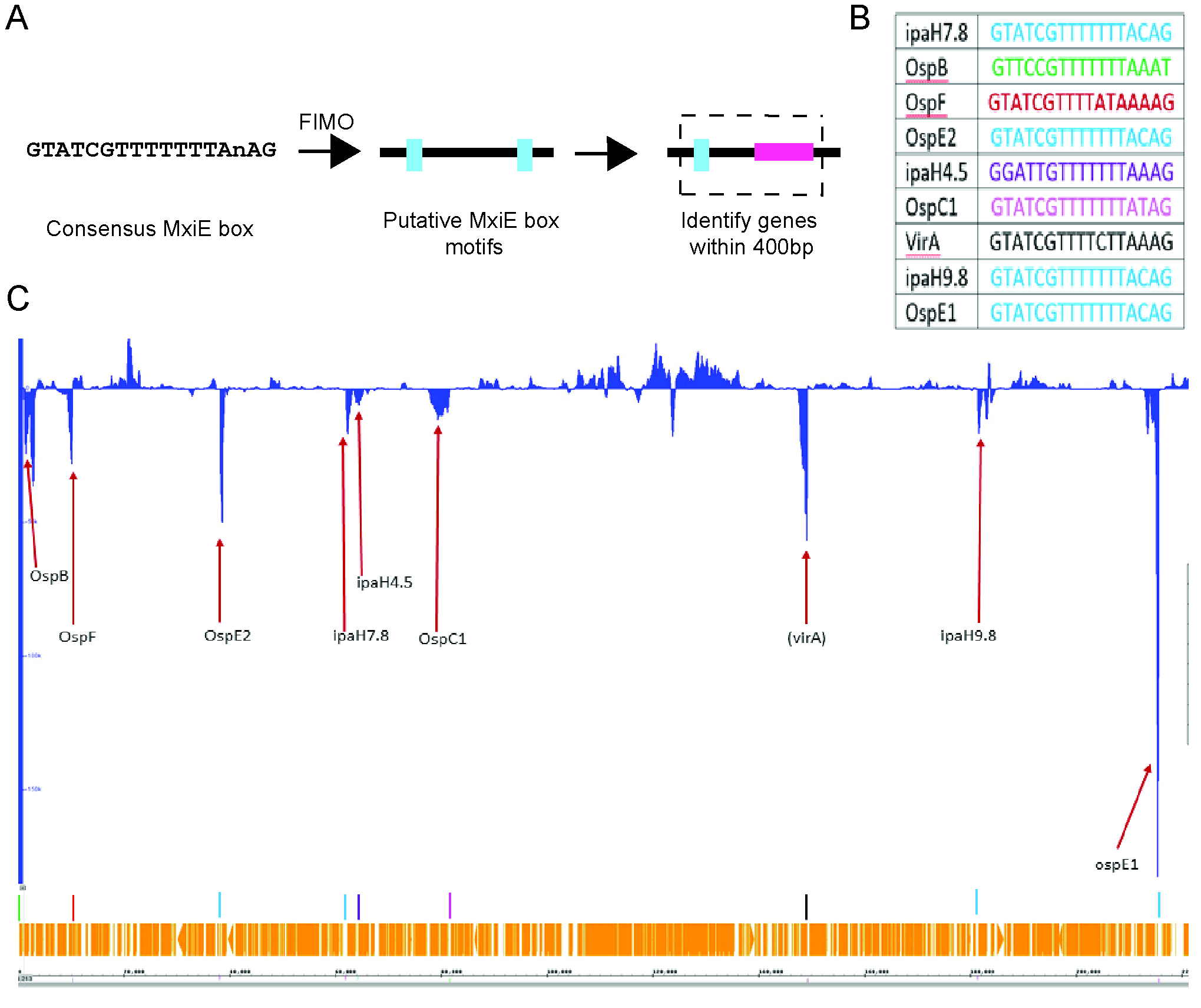

Supplement: FIG S1 [file msphere.00485-22-s0004.tif]

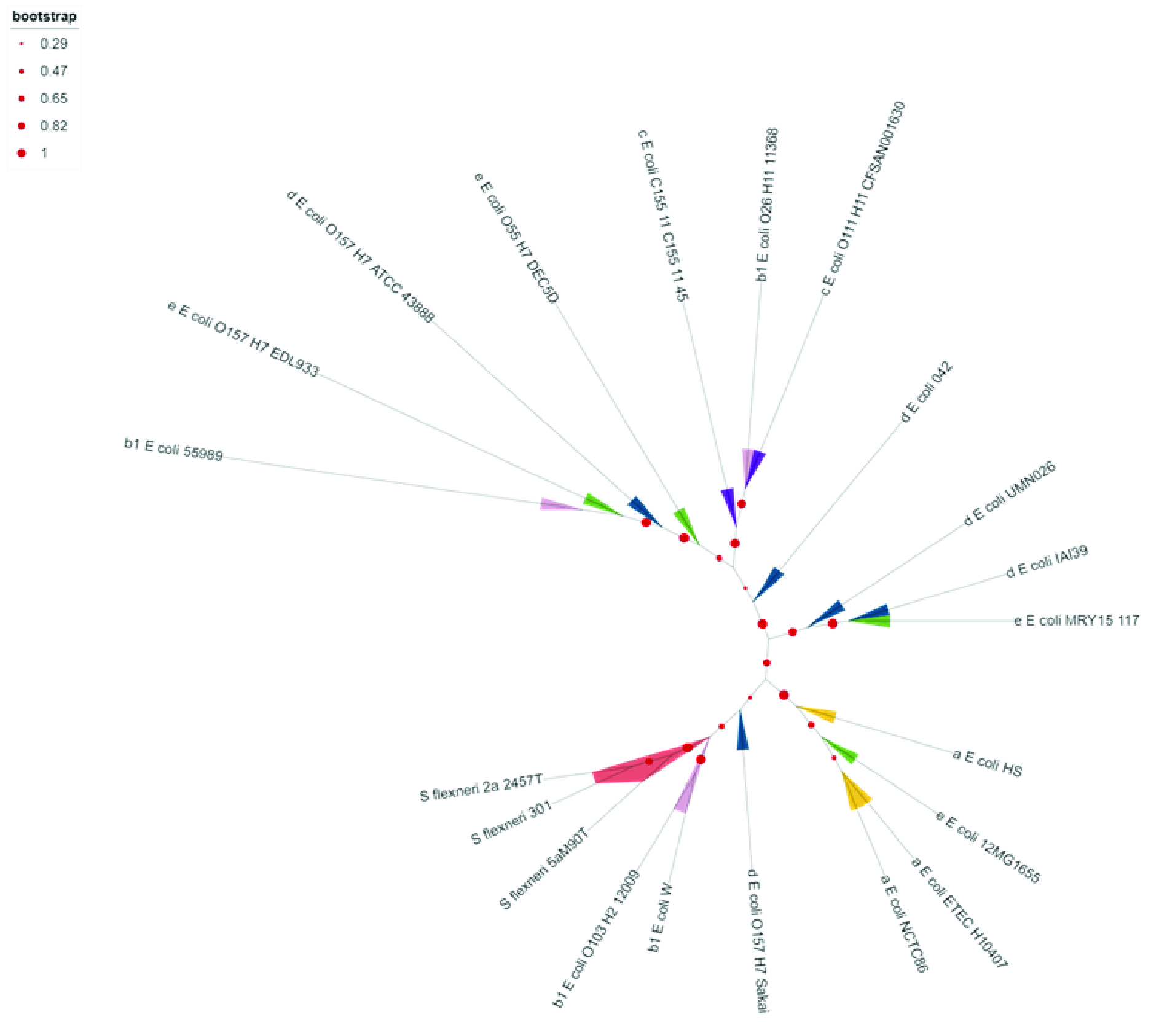

Supplement: FIG S2 [file msphere.00485-22-s0005.tif]

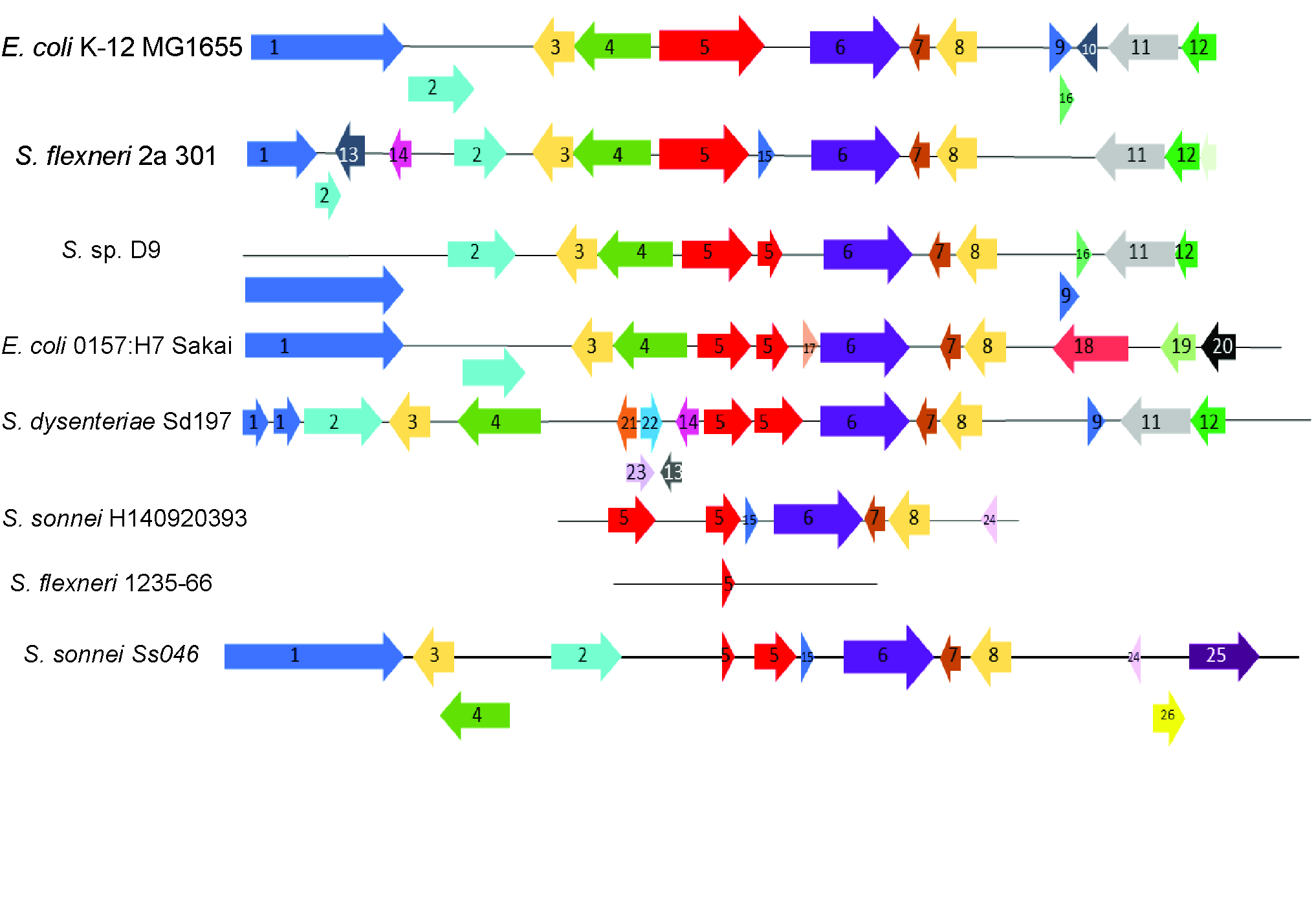

Supplement: FIG S3 [file msphere.00485-22-s0006.tif]

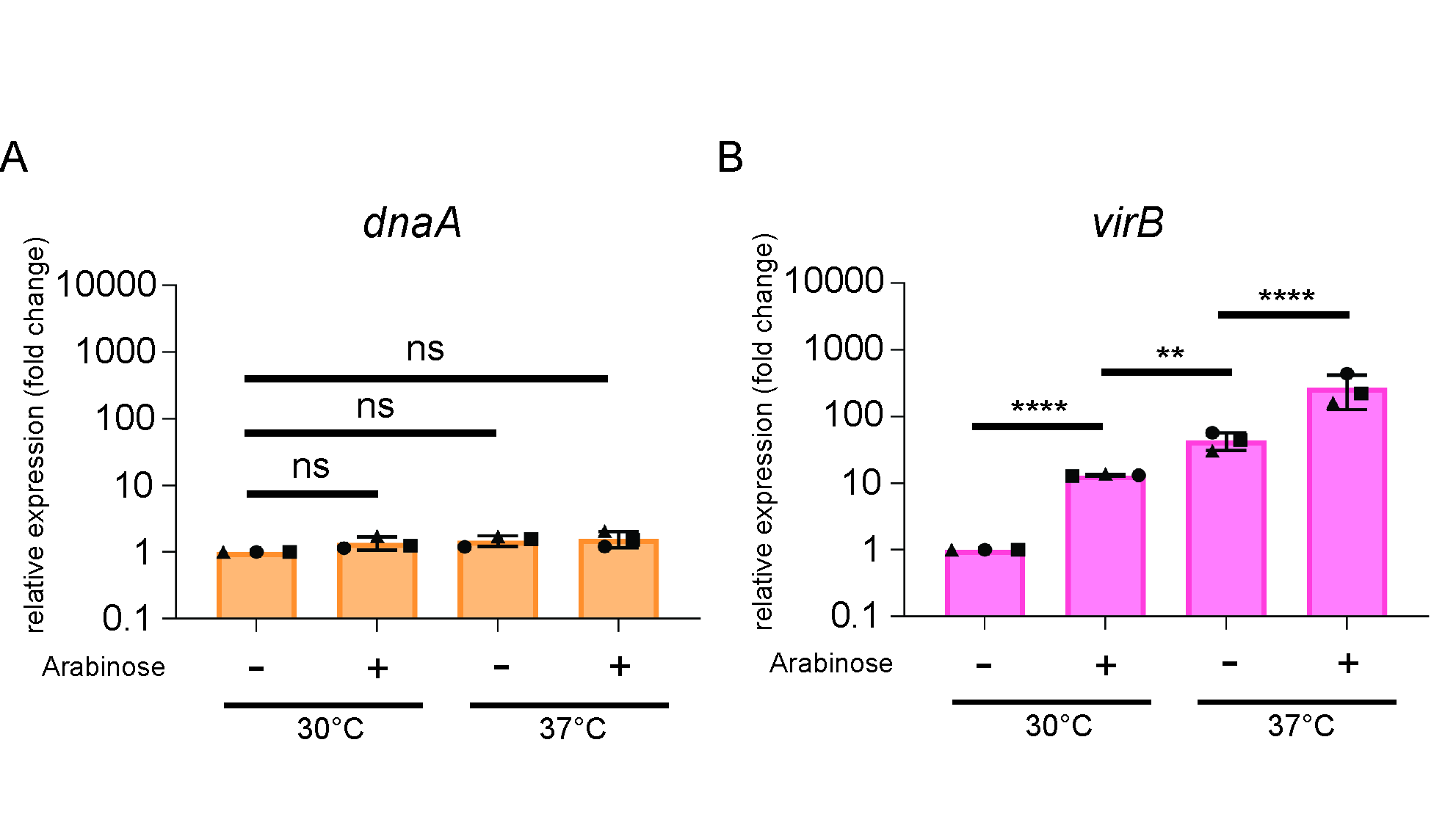

Supplement: FIG S4 [file msphere.00485-22-s0007.tif]

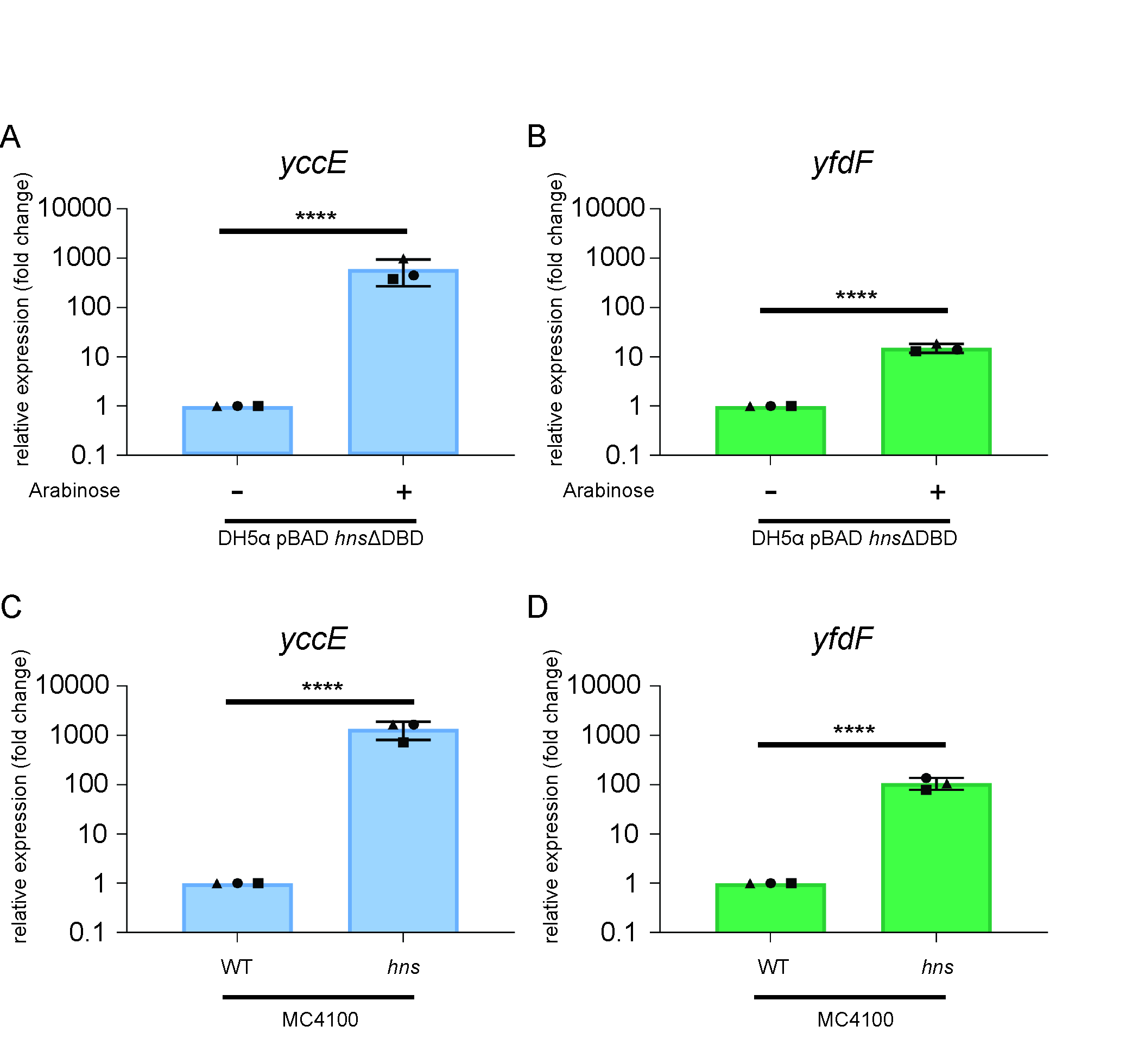

Supplement: FIG S5 [file msphere.00485-22-s0008.tif]
